# Case Report: Identification of a rare nonsense mutation in the *POC1A* gene by NGS in a diabetes mellitus patient

**DOI:** 10.3389/fgene.2023.1113314

**Published:** 2023-03-28

**Authors:** Dongfeng Li, Shihui Li, Jingjing Zhou, Lili Zheng, Gui Liu, Chengzhang Ding, Xingyun Yuan

**Affiliations:** Department of Endocrinology, Affiliated Hospital of West Anhui Health Vocational College, Lu’an, China

**Keywords:** special type of diabetes mellitus, POC1A, gene mutation, SOFT syndrome, insulin resistance

## Abstract

**Objective:** This study aimed to investigate the clinical and molecular biology of a patient with a type of diabetes mellitus caused by a mutation in the *POC1A* (OMIM number: 614783) gene and explore its pathogenesis and related characteristics.

**Methods:** The patient was interviewed about his medical history and subjected to relevant examinations. Blood DNA samples were collected from the patient and his family members (parents) for trio whole-exome sequencing. Whole-exome sequencing was performed using the IDT xGen Exome Research Panel v1.0 whole-exome capture chip and sequenced using an Illumina NovaSeq 6,000 series sequencer (PE150); the sequencing coverage of the target sequence was not less than 99%. After systematic analysis and screening of the cloud platform for accurate diagnosis of genetic diseases, which integrated molecular biology annotation, biology, genetics, and clinical feature analysis, combined with a pathogenic mutation database, normal human genome database, and clinical feature database of 4,000 known genetic diseases, hundreds of thousands of gene variants were graded using the gene data analysis algorithm, a three-element grading system, and the American Society of Medical Genetics gene variant grading system. After polymerase chain reaction testing, the target sequence was verified by Sanger sequencing using an ABI3730 sequencer, and the verification result was obtained using sequence analysis software.

**Results:** The patient had a peculiar face, a thin body, and a body mass index of 16.0 kg/m^2^. His fasting connecting peptide was 10.2 ug/L, his fasting insulin was 44 mIU/L, his fasting blood glucose was 10.5 mmol/L, and his glycosylated haemoglobin was 12.5%. After hospitalisation, the patient was given 0.75 g/d metformin tablets and 15 mg/d pioglitazone dispersible tablets, and his fasting blood glucose reduced to 9.2 mmol/L. After 48 U/L insulin treatment, the patient’s fasting blood glucose was reduced to 8.5 mmol/L. Genetic screening revealed that there was a pathogenic variant at the *POC1A* gene locus and a cytosine-to-thymine mutation at the G81 locus, turning the Arg to a termination codon and shortening the POC1A protein from 359 amino acids (aa) to 80 aa. No mutation was detected in the patient’s parents’ *POC1A* gene loci.

**Conclusion:** The patient’s diabetes was caused by a *POC1A* gene mutation at the G81 locus, which is rarely reported in the clinic. The specific manifestations of this mutation need to be further investigated.

## 1 Introduction

Diabetes mellitus has a clear aetiology and well-known characteristics. There are currently eight major types of diabetes, including type A insulin resistance syndrome (U.S. National Library of Medicine Genetics Home Reference, 2019), Donohue syndrome (also known as leprechaunism) (Huggard et al., 2015), Rabson–Mendenhall syndrome (Musso et al., 2004), and lipoatrophic diabetes (Lawson, 2009). The present study investigated a 32-year-old patient with a rare *POC1A* gene mutation resulting in a type of diabetes mellitus, for which a detailed clinical diagnosis and molecular biological analysis were performed.

## 2 Case description

### 2.1 Patient information

The patient was a 32-year-old male, unmarried, with elevated blood glucose (fasting blood glucose up to 15.0 mmol/L), who had been diagnosed with diabetes mellitus during hospitalisation for trauma a year ago. He was treated with premixed insulin, which he later discontinued himself due to poor glycaemic control, and irregular oral medication to lower glucose, with poor glycaemic control. On 15 August 2020, the patient was admitted to the hospital to adjust his blood glucose after a fasting blood glucose level of 18.62 mmol/L was detected during the examination for a proposed surgical treatment of ischial tuberosity. He had no complaint of hypoglycaemia since the onset of the disease. He had normal growth and development in early childhood, but his growth slowed down at about 6 years old and stopped at about 10 years old. He had no history of other chronic or infectious diseases and no family history of hereditary diseases. He had a history of smoking 15 cigarettes a day for 10 years. His parents were consanguineous, and both were healthy, with normal random blood glucose and no similar symptoms. His grandparents had no history of diabetes.

### 2.2 Relevant physical examination

**FIGURE 1:**
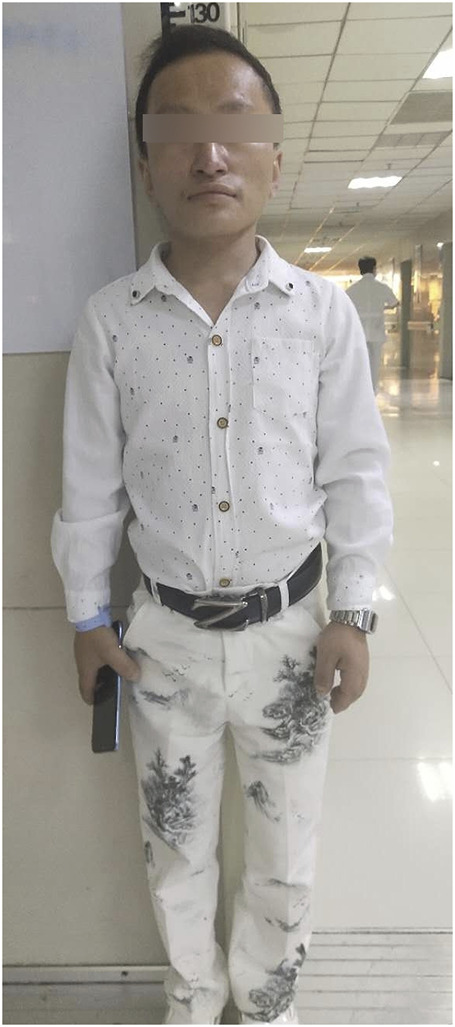
Full body photo.

Physical examination revealed that the patient had poor growth and development, being thin and with short stature. His height was 125 cm, his weight was 25 kg, and he had a body mass index of 16.0 kg/m2. His facial features included a macrocephaly (head circumference 58 cm), bossing of the forehead, prognathism, prominent nose, and thin hair. There were low set ear, hyperlipidemia and no significant deepening of the skin colour on his body and no significant black acanthoid changes on his neck, armpits, or popliteal fossa. His extremities were small, and the phalanges of both his hands were thick and short. A mass (3 cm × 3 cm) was palpable in his left buttock. Secondary sexual characteristics were developed, the external genitalia were slightly immature, and he had little pubic hair. His primary hearing screening was normal (see Figure 1) as the result of auditory brain response (ABR) examination by MEB-9404C machine.

**FIGURE 2:**
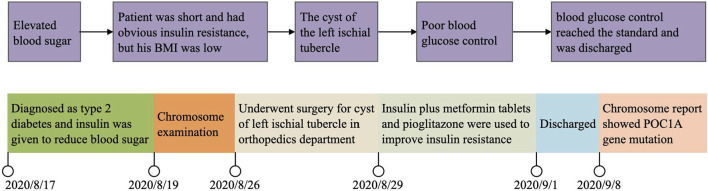
Showcasing a timeline with relevant data from the episode of care.

A figure showcasing a timeline with relevant data from the episode of care (see Figure 2).

## 3 Diagnostic assessment and outcomes

**FIGURE 3:**
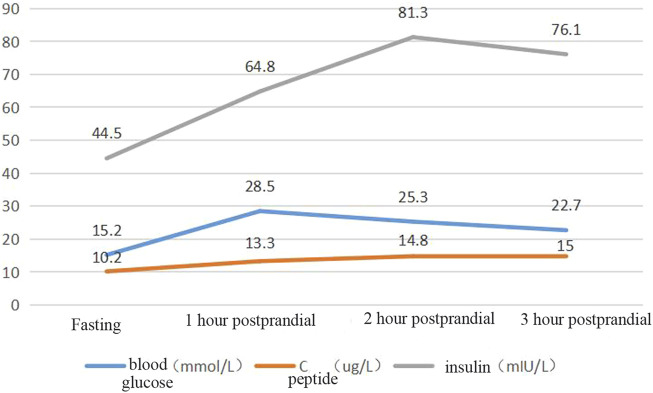
Insulin and C-peptide release test.

Blood glucose, insulin, and related antibody tests were performed after admission. The results showed mild liver function damage, normal thyroid function, a normal routine blood test, a fasting blood glucose of 15.02 mmol/L, and glycosylated haemoglobin of 12.5%. The patient’s urine glucose was 4+ and negative for diabetes autoantibodies (ICA, IAA, GADA). Colour Doppler ultrasound showed a fatty liver. The patient’s insulin and connecting peptide (C-peptide) release test results are shown in Figure 3.

**FIGURE 4:**
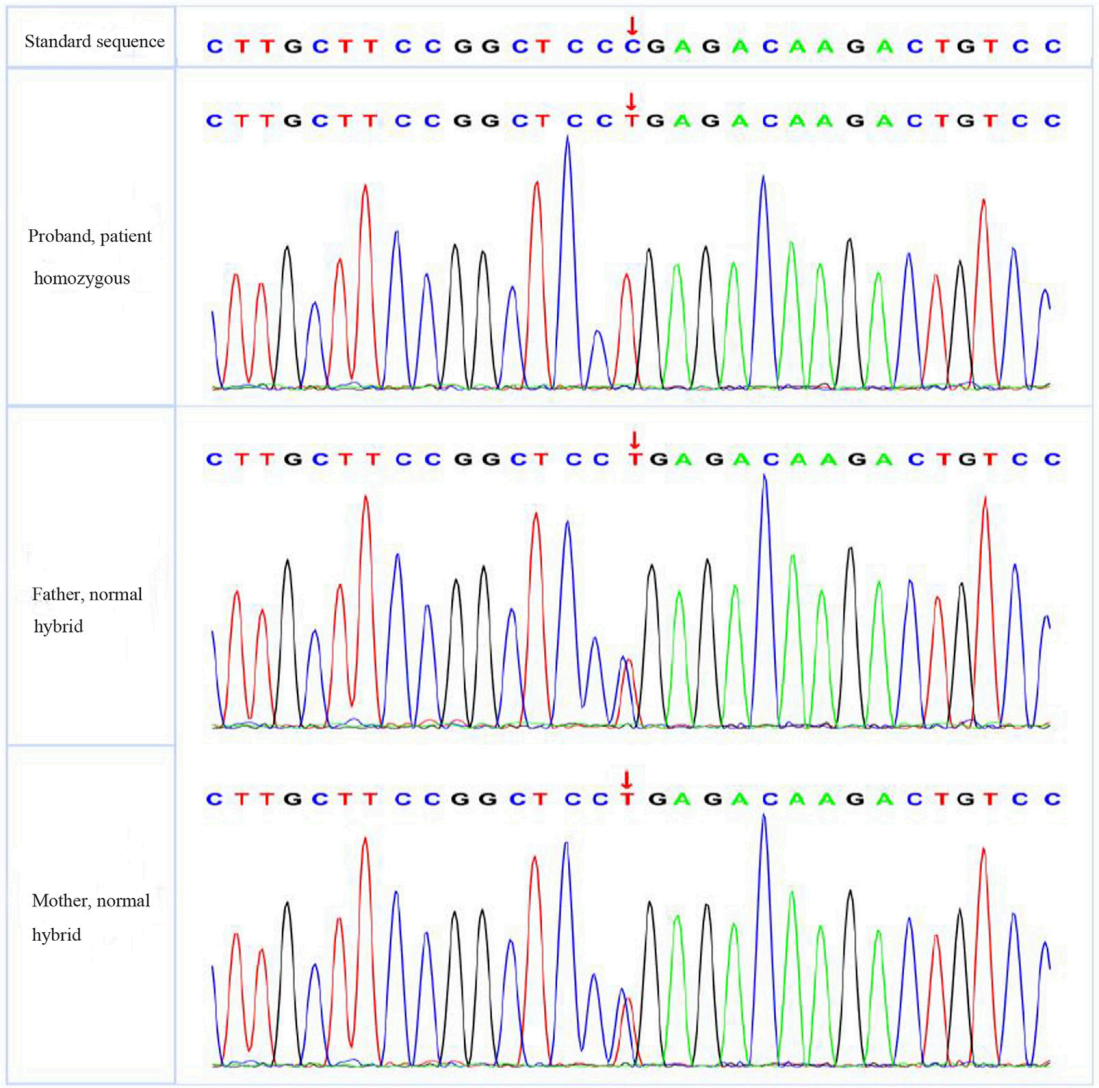
Sequencing results of PCR products of the proband and his family members.

The patient had significantly elevated blood glucose but did not rely on insulin therapy and did not have signs of ketoacidosis. He was negative for insulin-related antibodies and was found to have high C-peptide and insulin levels during the inpatient examination, so type 1 diabetes was excluded. The patient was treated with a high dose of insulin and was judged to be insulin resistant on the basis of the results of his insulin and C-peptide release tests. Based on the patient’s short stature, facial features, and parental consanguinity history, a particular type of diabetes needed to be considered for diagnosis, and gene sequencing was performed. A total of 3 mL of whole peripheral blood was extracted from three members of the family (the proband, his father, and his mother) for trio whole-exome sequencing. The results showed that the patient had a pathogenic *POC1A* (NM_015426.5) gene variation; specifically, the base at position 241 in the coding region was a cytosine-to-thymine (C-to-T) mutation, and this nonsense mutation was located in exon 3. As a result, the encoded protein was terminated in advance at amino acid 81, shortening the 327 amino acids (c.241(exon 3) C>T, p.R81X,327) (see Figure 4).The associated diseases were short stature, nail hypoplasia, facial dysmorphism, hypotrichosis, all of which are associated with SOFT syndrome (clinically characterised by diabetes, short stature, and phalangeal abnormalities in the fingers). And the phalanges we detected in this study of both hands were thick and short. In combination with the patient’s clinical features, the diagnosis of a specific type of diabetes mellitus (SOFT syndrome) was confirmed. The proband was homozygous, and his parents were heterozygous, which is consistent with the pathogenesis of autosomal recessive disorders.

## 4 Discussion

There are many substitution–nonsense mutations in the POC1A gene has been reported before (Sjöblom et al., 2006; Cancer Genome Atlas Network, 2012; George et al., 2015; Landau et al., 2015; Robinson et al., 2015; Hayward et al., 2017; Newell et al., 2019; Lawson et al., 2020). In the COSMIC database, 267 variants are reported in this gene, the mutation in this case, in c.241C>T, p.R81* COSM4716289; is also reported in three samples, and another variant of this also reported in c.242G>A; p.R81Q, COSM3775216 in one sample. In 2012, Shalev et al. (Shalev et al., 2012) first reported an autosomal recessive disorder caused by mutations in the PCO1A gene that was characterised by short stature, nail hypoplasia, facial dysmorphism, and hypotrichosis. Subsequently, Sarig (Sarig et al., 2012) reported such cases and applied the term ‘SOFT syndrome’. To date, 29 cases of SOFT syndrome have been reported worldwide, with only one case being reported in China (Li et al., 2021). According to current reports, patients with SOFT syndrome present with various manifestations, including macrocephaly, microcephaly, low-set ears, hypertrichosis, hyperlipidaemia, acanthosis nigricans, and severe insulin resistance, in addition to the more common features (Al-Kindi et al., 2020).

Most patients diagnosed with SOFT syndrome have acanthosis nigricans and severe insulin resistance, but they are usually diagnosed with dwarfism rather than diabetes before puberty, at which stage their glycaemic values have not yet reached the criteria for diabetes mellitus. In the present study, a 32-year-old male patient was initially diagnosed with type 2 diabetes mellitus and primary dwarfism. Due to the patient’s facial features, thinning hair, severe insulin resistance, and parents’ consanguineous marriage, the possibility of a genetic mutation was put forward. The results of subsequent genetic testing confirmed a pathogenic mutation at the *POC1A* G81 gene locus.

Centrioles are important structures found in most animal cells. They consist of nine highly stable microtubule triads that form a barrel-shaped blade about 500 nm long and 200 nm in diameter (Azimzadeh and Marshall, 2010). Centrioles are thought to play an essential role in human mitosis and cilia formation, which are associated with human diseases, especially developmental abnormalities. The proteome of centrioles 1 (POC1) is a core component of centrioles and includes two different isoforms, POC1A and POC1B, which are encoded by different genes. POC1 is mainly involved in centriole replication and length control, as well as ciliogenesis (Venoux et al., 2013). POC1A is localised on the inner wall of centrioles, is proximal to them, and plays a role in centriole formation and maintenance (Venoux et al., 2013). Mutations in POC1A can lead to abnormal mitotic spindles and centrioles, which severely impair centriole function and cause disease (Shaheen et al., 2012). Spermatogonial stem cell transplantation studies have shown that POC1A is essential for the normal function of Sertoli cells and germ cells and that POC1A mutations can lead to skeletal dysplasia and primary dwarfism (Geister et al., 2015).

Most of the currently reported *POC1A* gene mutations in SOFT syndrome occur in exons 6, 8, 9, and 10. Three of these cases, all of which exhibit short stature, hyperlipidaemia, acanthosis nigricans, and extreme insulin resistance with highly overlapping clinical features, have been found to have homozygous frameshift mutations in exon 10 of the *POC1A* gene (Azimzadeh and Marshall, 2010). Thus, Giorgio (Azimzadeh and Marshall, 2010) suggested that *POC1A* gene mutations can lead to two different conditions: SOFT syndrome and variant POC1A (vPOC1A) syndrome. The primary clinical differences between these two phenotypes are extreme dyslipidaemia with insulin resistance and acanthosis nigricans, which are the main features of vPOC1A syndrome but do not appear in SOFT syndrome. A novel homozygous frameshift mutation in the patient’s POC1A may affect two of the three protein products of this gene, which are associated with excess centrioles and multipolar mitotic spindles in primary cells with extreme dyslipidaemia and insulin resistance (Majore et al., 2021). *POC1A* encodes a protein associated with centrioles throughout the cell cycle and is involved in the mitotic spindle and primary cilia function; its dysfunction leads to extreme dyslipidaemia and insulin resistance (Majore et al., 2021).

The mutation in the *POC1A* gene, c.241C>T, is not consistent with any previously reported cases. However, only one case of gene variation at this locus has been identified, and further studies are needed to explore its specific pathogenesis. A timely and accurate genetic diagnosis of such patients with specific clinical manifestations of diabetes would help clinicians select appropriate treatments and predict and prevent the development of the disease in family members.

## Data Availability

The datasets presented in this article are not readily available because of restrictions on the release of the data imposed by Affiliated Hospital of West Anhui Health Vocational College. Requests to access the datasets should be directed to corresponding author DL (dongfengli81@163.com).
